# Pneumatic spray delivery‐based solid set canopy delivery system for oblique banded leaf roller and codling moth control in a high‐density modern apple orchard

**DOI:** 10.1002/ps.7099

**Published:** 2022-08-13

**Authors:** Ramesh K. Sahni, Rakesh Ranjan, Gwen‐Alyn Hoheisel, Lav R. Khot, Elizabeth H. Beers, Matthew J. Grieshop

**Affiliations:** ^1^ Center for Precision and Automated Agricultural Systems Washington State University Prosser WA USA; ^2^ Department of Biological Systems Engineering Washington State University Prosser WA USA; ^3^ Freshwater Institute The Conservation Fund Shepherdstown WV USA; ^4^ Department of Entomology Washington State University Wenatchee WA USA; ^5^ The Grimm Family Center for Organic Production and Research Cal Poly San Luis Obispo CA USA

**Keywords:** fixed spray delivery, pneumatic spray delivery, biological efficacy, leaf bioassay, fruit bioassay, larval mortality

## Abstract

**BACKGROUND:**

Pneumatic spray delivery (PSD)‐based solid set canopy delivery systems (SSCDS) have demonstrated comparable spray deposition and reduced off‐target drift compared with axial‐fan airblast sprayers in high‐density apple orchards. An important next step is to quantify whether PSD‐based SSCDS can provide effective pest management. This study evaluated the biological efficacy of this fixed spray system variant and compared it with that of an axial‐fan airblast sprayer. Partial field trials were conducted in a commercial apple orchard (*cv.* Jazz) trained in tall spindle architecture. Insecticides were applied at a rate of 935 L ha^−1^ (100 gallons per acre) for both application systems. Twenty‐four hours after spraying, leaves and fruits were collected to prepare the laboratory bioassays enabling evaluation of efficacy against obliquebanded leafroller (OBLR) and codling moth (CM).

**RESULTS:**

OBLR mortality for SSCDS, airblast sprayer and untreated control treatments after 24 h of larval exposure was 91%, 98% and 4%, respectively and increased to 98%, 100% and 19% after 48 h. First‐instar CM leaf bioassay mortality was 100% for SSCDS and airblast sprayer treatment, and 13% for the untreated control at 24 h post exposure. Larval CM mortality on fruit was 100% for SSCDS and airblast sprayer treatments, and 33% on the untreated control.

**CONCLUSIONS:**

Insecticides applied using SSCDS and an airblast sprayer had comparable larval mortality in all three assays, significantly higher than the untreated controls. These results suggest that PSD‐based SSCDS may provide a viable alternative to axial‐fan airblast sprayers in high‐density apple orchards. © 2022 The Authors. *Pest Management Science* published by John Wiley & Sons Ltd on behalf of Society of Chemical Industry.

## Graphical abstract

Pneumatic spray delivery‐based solid set canopy delivery systems and an airblast sprayer demonstrated comparable larval mortality for obliquebanded leafroller in leaf bioassay and codling moth in leaf and fruit bioassays.

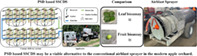

## 1 INTRODUCTION

Washington (WA) state produces 3.8 million tons of apples annually, totaling 69% of fresh market production in the United States.^1^ Apple growers in this region face considerable fruit damage and related economic losses due to arthropod pest infestations.^2^ The most serious insects and diseases affecting apple crops in this region include codling moth, leafrollers, apple maggot, aphids, fire blight and powdery mildew.^3^, ^4^ These complexes, if not managed properly, can adversely affect the crop yield and result in suboptimal fruit quality. Codling moth (CM, *Cydia pomonella*) and obliquebanded leafroller (OBLR, *Choristoneura rosaceana* (Harris)) are the most damaging insects infesting apples in WA state.^5^, ^6^ CM larvae feed on the fruit internally and make it unmarketable.^7^ Similarly, OBLR feeds on the foliage and fruits close to foliage causing significant fruit damage.^6^, ^8^

Airblast sprayers are the most common technology used for applying crop protectants to apples in the United States and WA state.^9^, ^10^ Although these sprayers were initially designed for traditional tall, spherical canopies, most apple orchards have transitioned to high‐density, linear canopy architectures (for example, tall spindle, v‐trellis and bi‐axis). The purpose of this transition was to achieve higher labor efficiency for orchard management and maximize fruit production and quality.^11^, ^12^ The use of airblast sprayers in high‐density systems has consistently been shown to cause excessive off‐target spray drift.^13^, ^14^, ^15^, ^16^, ^17^, ^18^, ^19^, ^20^ Drift results in environmental and natural ecosystem contamination and increases the risk of community exposure to harmful chemicals.^21^, ^22^, ^23^, ^24^ As an alternative, researchers have been exploring fixed spray systems most recently called solid set canopy delivery systems (SSCDS).^19^, ^25^, ^26^, ^27^, ^28^, ^29^, ^30^, ^31^, ^32^, ^33^, ^34^, ^35^, ^36^, ^37^, ^38^, ^39^, ^40^, ^41^, ^42^

SSCDS have been extensively tested and optimized for performance in terms of spray deposition and coverage.^19^, ^31^, ^32^, ^33^, ^34^, ^37^, ^38^, ^39^, ^40^, ^41^, ^42^ However, limited information is available on the biological efficacy of such systems for insect and pest management. A small orchard‐scale study was conducted in Prigonrieux (France), targeting apple scab management.^30^ This study reported similar scab control compared with an airblast sprayer. A similar study was conducted in Québec (Canada) to evaluate the efficacy of an overhead fixed spray delivery system against two conventional airblast sprayer variants for apple scab management in a modern orchard.^29^ The study reported comparable performance of a fixed spray delivery system against a conventional airblast spray system in terms of apple scab control. A study evaluating SSCDS and axial‐fan airblast sprayer applications for apple scab and arthropods (OBLR, stinkbug, CM, oriental moth and plum curculio) management was conducted in Michigan (USA) apple orchards.^36^ For airblast sprayer‐ and SSCDS‐treated plots, scab incidence was observed in the range of 0%–2% compared with untreated control plots which had 80% incidence. Total fruit damage due to arthropod infestation post SSCDS and airblast sprayer treatments was 4% and was statistically equivalent. The efficacy of SSCDS was also evaluated through leaf bioassay on OBLR larvae.^35^ Results indicated a non‐significant difference in OBLR larval mortality for the SSCDS and airblast sprayer with mortality of 95% and 98%, respectively. Furthermore, field fruit damage evaluations did not indicate any significant differences between the airblast and SSCDS treatments with minimal fruit damage.

Our group has extensively researched SSCDS configurations for tall spindle apple architectures typical in WA state.^38^, ^40^, ^41^, ^42^, ^43^, ^44^ PSD‐based SSCDS developed by Sinha *et al*. provided improved spray deposition and uniform spray discharge along the spray line compared with hydraulic‐based SSCDS^38^. Our latest optimal PSD‐based SSCDS provided similar spray deposition, reduced ground drift and negligible aerial drift, but lower spray coverage compared with an airblast sprayer in tall spindle apple orchard.^44^ This system has not yet been validated for biological efficacy.

Demonstrating biological efficacy data specific to important WA state insects for fixed spray systems with respect to airblast spray applications is critical to support large‐scale commercial system adoption. Thus, this study was conducted to investigate the biological efficacy of optimized PSD‐based SSCDS^44^ for control of OBLR and CM in a commercial apple orchard trained in tall spindle apple architectures typical of WA state. Biological efficacy was evaluated in terms of larval mortality through leaf and fruit bioassays. Mortality results from insecticides applied using PSD‐based SSCDS were compared with that of axial‐fan airblast sprayer applications and untreated control.

## 2 MATERIALS AND METHODS

### 2.1 Experimental site

**Figure 1:**
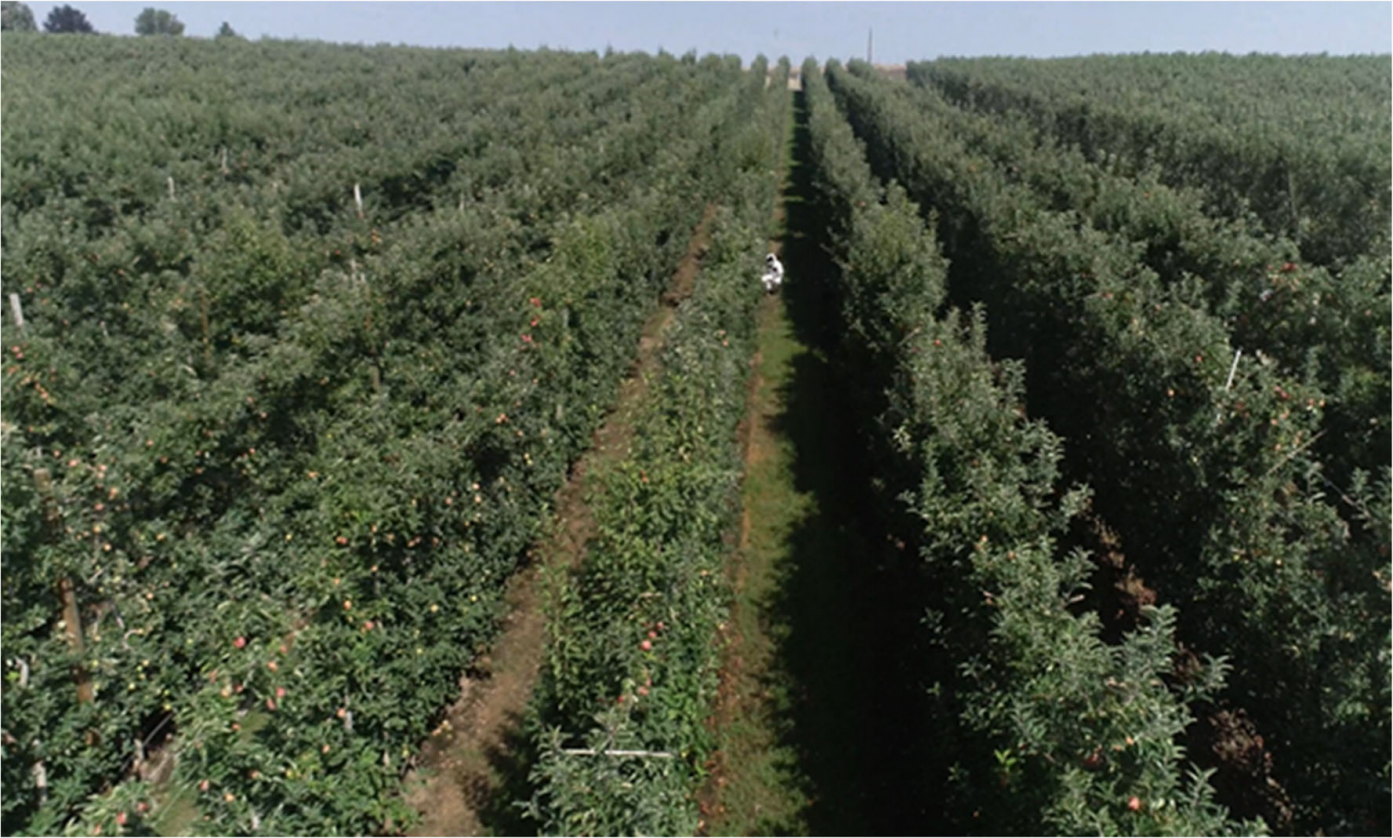
Orchard site selected for the biological efficacy study

A commercial apple orchard (*cv*. Jazz; 11.91 ha) (Figure 1) located in Grandview, WA (46.309°N, 119.844°W, 393 m a.s.l.) was selected for the experiment. The apple trees in the orchard were planted in 2006 and grafted on a NIC 29‐EMLA 9 rootstock. The trees were trained in a modern tall spindle architecture supported on an eight‐wire trellis system. Tree rows were oriented in north–south direction with row spacing of 2.74 m, tree spacing of 1.52 m, mean tree height of 3.66 m, and tree density of 2400 trees ha^−1^. Untreated control leaves were collected from a research orchard (*cv*. Virginia Crab; 0.10 ha) located at Irrigated Agricultural Research and Extension Center, Washington State University, Prosser, WA (46.255°N, 119.739°W, 267 m a.s.l.). This orchard had not received any pesticide application for the last 12 months.

### 2.2 Spray application systems

**Figure 2:**
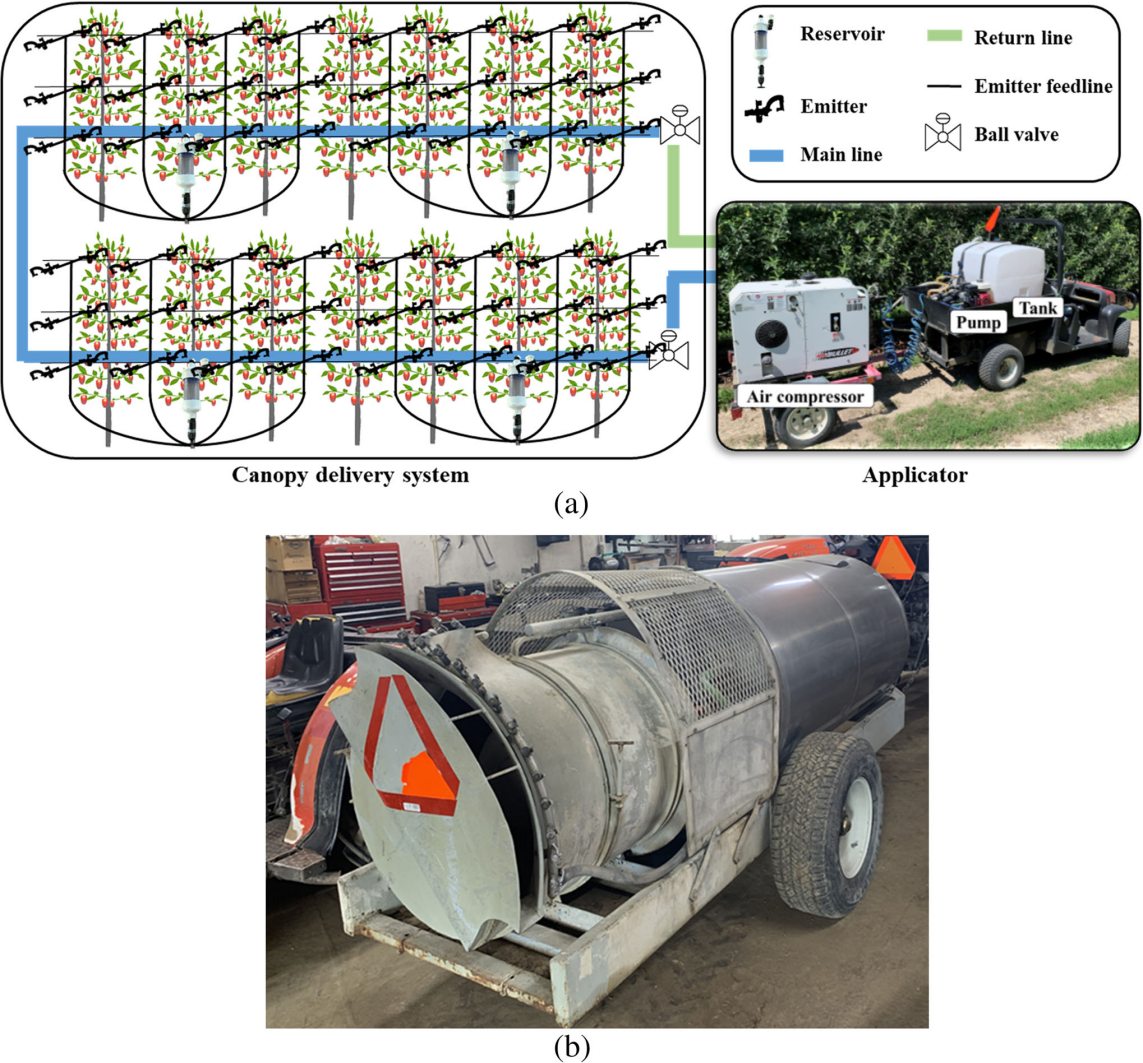
Schematics of (a) pneumatic spray delivery‐based solid set canopy delivery system with reservoirs to supply a metered spray liquid volume to emitter assemblies arranged in a three‐tier configuration, and (b) airblast sprayer used in this study.

The biological efficacy of two spray application systems, a PSD‐based SSCDS and an axial‐fan airblast sprayer, were evaluated. The configurational and operational details of SSCDS used in this study can be found in Sahni *et al*.^44^ Briefly, SSCDS is comprised of an applicator and a canopy delivery system (Figure 2a). The applicator consists of a centrifugal pump (1538, Hypro; flow rate of 0.17 m^3^ min^−1^ at 345 kPa), an air compressor (Bullet G 70, Boss Industries; flow rate of 1.98 m^3^ min^−1^ at 689.5 kPa), and a storage tank (379 L volumetric capacity). The canopy delivery system consists of spray lines (main and return), reservoirs (740 ml unit volumetric capacity), emitter feedline and emitter assembly. The spray lines were installed on the existing trellis wire at 0.63 m above ground level (a.g.l.) and connected in a loop with a manual ball valve at the start and end of the loop. The spray lines in the adjacent rows were looped to develop a 77‐m spray line. Each emitter assembly consists of a pair of micro‐emitters (Modular 7000, Jain Irrigation; flow rate of 0.66 L min^−1^ at 310 kPa) installed 0.6 m apart on a bamboo stick. These assemblies were installed in three‐tier arrangement in the bottom (1 m a.g.l.), mid (2.1 m a.g.l.) and top (3 m a.g.l.) canopy zone. The reservoirs were installed at a spacing of 6.1 m and connected to four emitter feedlines that supply spray liquid to the emitter assemblies.

A commercial axial‐fan airblast sprayer (S30P400, Heavy Duty, Turbo‐Mist; ten blades; 0.76 m fan diameter; tank capacity of 1514 L) (Figure 2b) was used in the other treatment. The sprayer was powered by power‐take‐off of an agriculture utility vehicle (M8540, Kubota Tractor Corp.; power‐take‐off = 56 kW). When observed from back of the sprayer, the airblast fan rotates in the counterclockwise direction. Per the grower's field consultant, 22 of the 26 spray nozzles (13 each side; hardened stainless steel with DC25 cores, TeeJet Technologies) (Table 1) were used to spray active canopy zones. The total flow rate for either side of the sprayer was 21.37 L min^−1^. Travelling at a forward speed of 1.34 m s^−1^ and operating pressure of 758 kPa, the airblast sprayer delivered 935 L ha^−1^ (100 gallons per acre).

**Table 1:** Summary of nozzle attributes of axial‐fan airblast sprayer

Nozzle position (from sprayer bottom)	Nozzle type	Nominal flow rate (L min^−1^)
13	D6 DC25	2.65
12	D6 DC25	2.65
11	D6 DC25	2.65
10	D5 DC25	2.04
9	D5 DC25	2.04
8	D5 DC25	2.04
7	D4 DC25	1.70
6	D4 DC25	1.70
5	D4 DC25	1.70
4	D3 DC25	1.10
3	D3 DC25	1.10
2^a^	—	—
1^a^	—	—

^a^ Inactivate nozzle.

### 2.3 Insect rearing

#### 2.3.1 Obliquebanded leafroller

**Figure 3:**
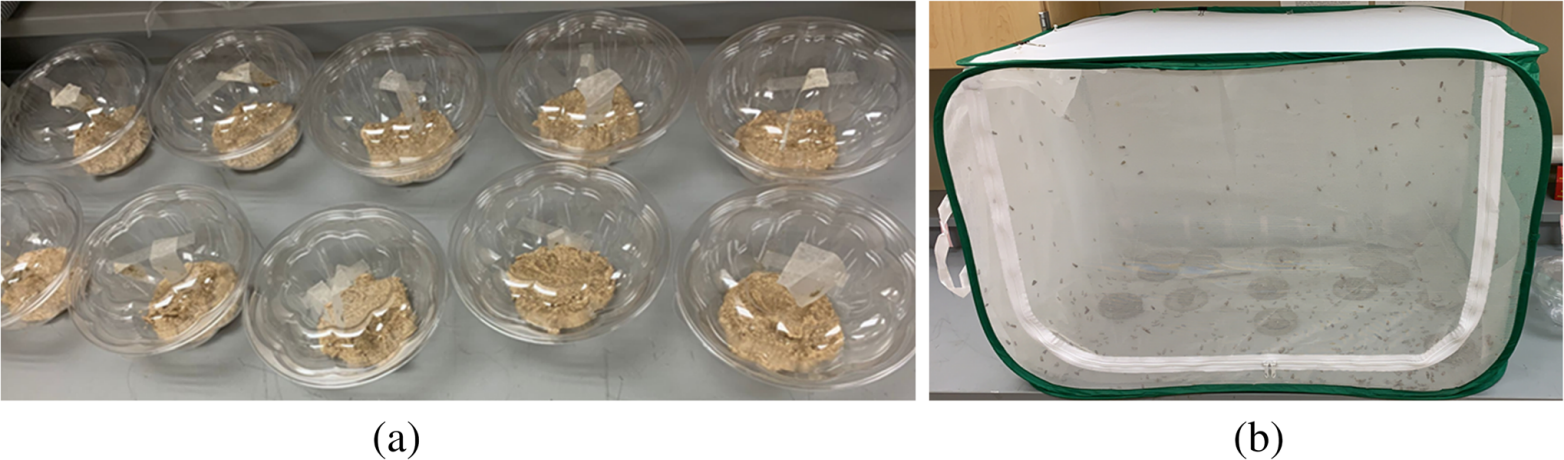
Rearing setup for (a) obliquebanded leafroller larvae fed on a pinto bean diet and (b) codling moth fed on a honey syrup‐based diet in the laboratory

OBLR egg masses were procured from the Trevor Nichols Research Center (Michigan State University). The egg masses (deposited on wax paper) were reared in the laboratory. The eggs were first held at room temperature (23 ± 2°C) until the dark head capsules of the larvae became visible. A pinto bean‐based artificial diet was prepared and transferred to ten plastic bowls (polyethylene terephthalate; top diameter, 17.5 cm; bottom diameter, 8 cm; height, 10 cm; volume, 0.95 L). Thereafter, wax paper sheets containing approximately 100 eggs were hung on the inner surface of the lid with the help of tape (Figure 3a) and the lid was closed. The lid was perforated with a pin to maintain limited air flow with high internal relative humidity and temperature. The bowls were stored at room temperature with a 16:8 h light/dark photoperiod. The larvae were reared until they reached the third instar, the stage used for the bioassays.

#### 2.3.2 Codling moth

Adult CM were procured from a laboratory (OK‐SIR rearing facility, Osoyoos, BC, Canada). The moths were kept in a screen cage (60 × 60 × 90 cm) at room temperature (23 ± 2°C) with a 16:8 h light/dark photoperiod (Figure 3b) and fed using a cotton ball saturated with 20% aqueous honey solution and placed in a Petri dish (diameter 9 cm) on the bottom of the cage. These dishes were replaced at an interval of 2 days.^45^ The inner surfaces of the cage were covered with wax paper strips to provide a substrate for oviposition. Wax paper strips with deposited eggs were replaced with new ones at three‐day intervals. Eggs masses were stored at room temperature in a covered plastic bowl until hatching. The first‐instar CM (neonates) were used for the experiments.

### 2.4 Experimental design and sample collection

**Figure 4:**
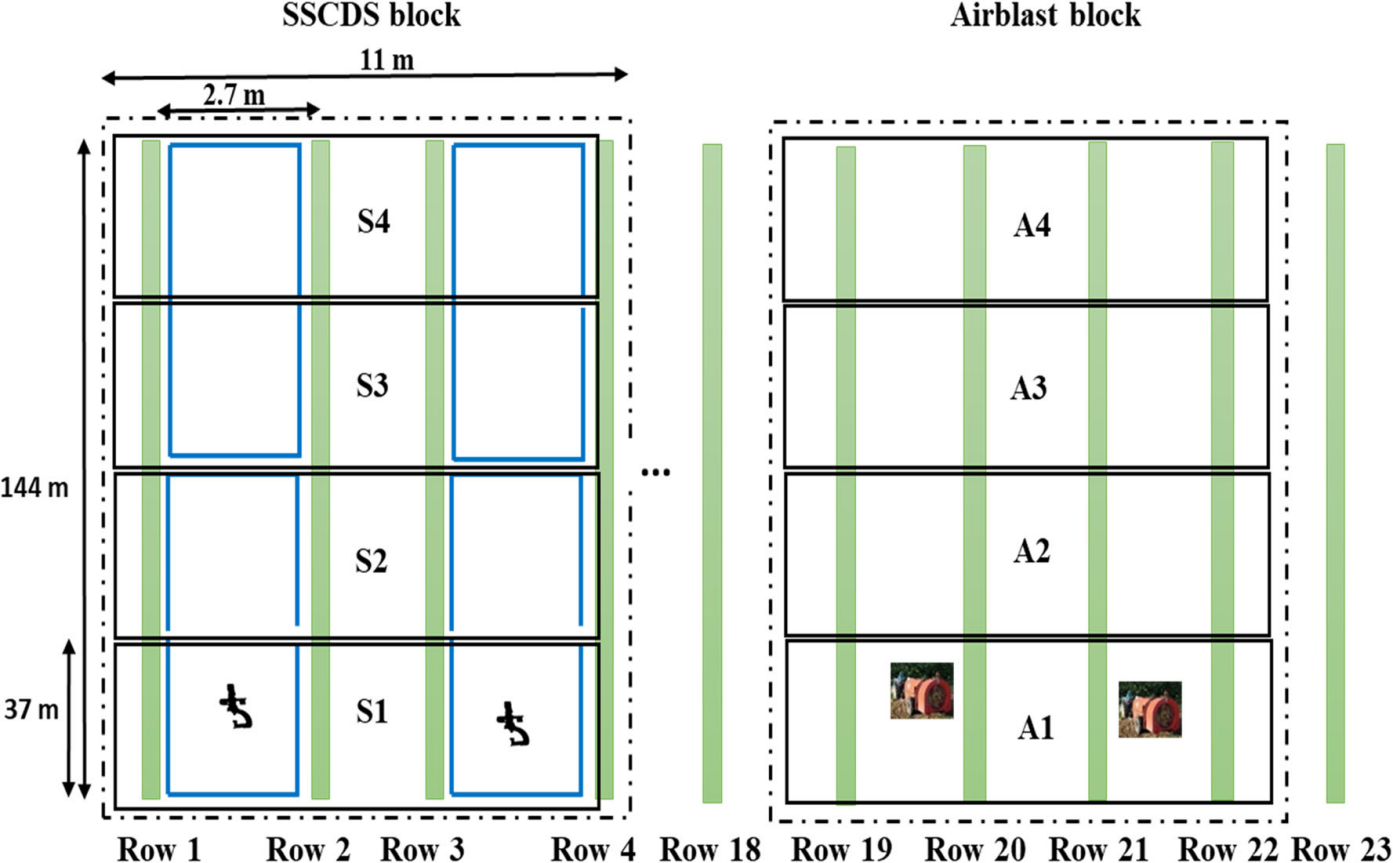
Experimental plot design for the biological efficacy trials on a solid set canopy delivery system (S1–S4) and airblast sprayer (A1–A4)

**Figure 5:**
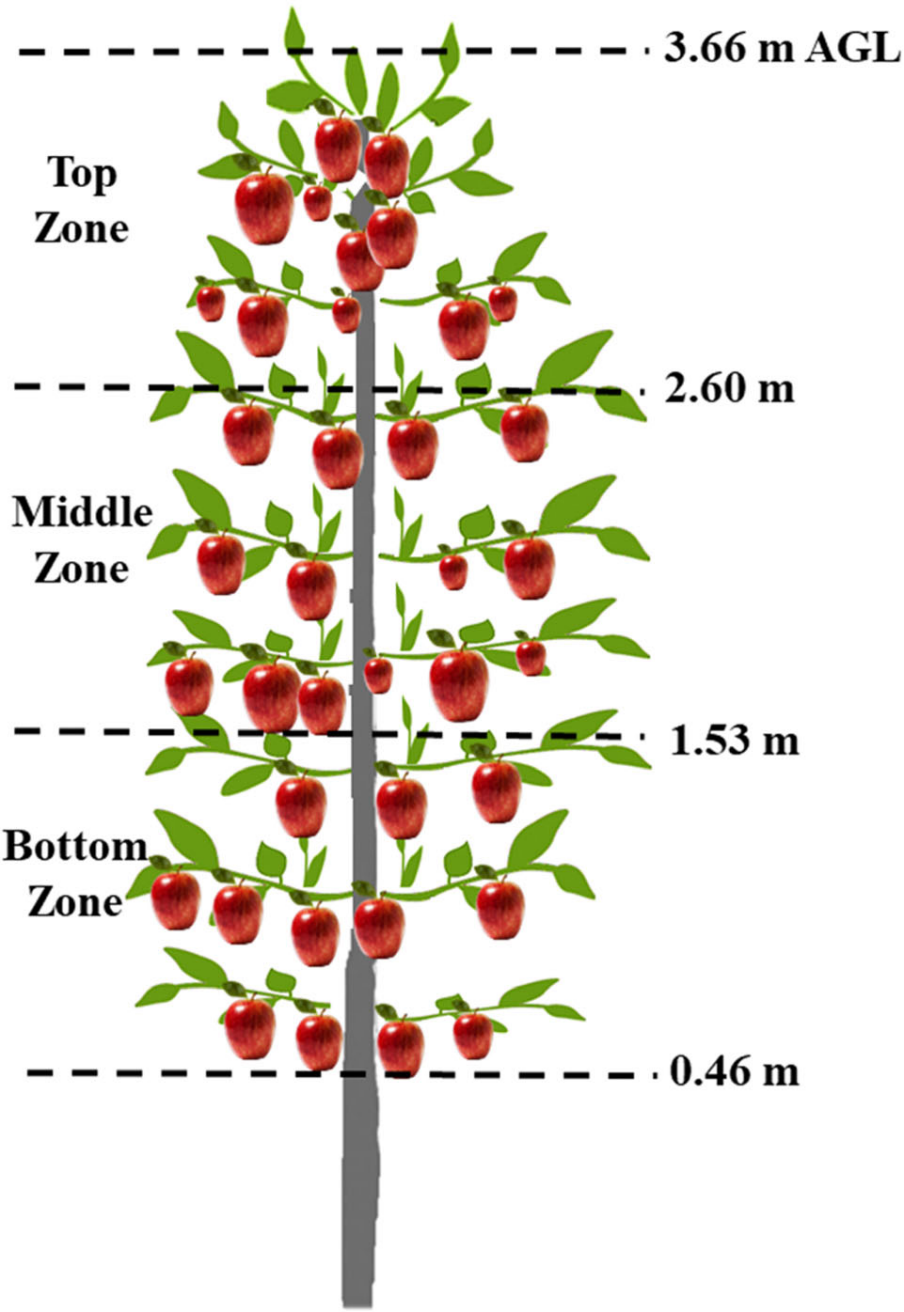
Schematic of sampling zone on the tree. All dimensions are in meters and are not to scale

Four consecutive tree rows (rows 1–4) were selected and divided into four plots (S1–S4, 0.04 ha [37 m × 11 m] and approximately 100 trees per plot) (Figure 4). Eight loops (77 m long) of PSD‐based SSCDS were installed in these plots as described in Sahni *et al*.^44^ Similarly, for the airblast sprayer treatment, four rows (rows 19–22) were equally divided into four plots (A1–A4, Figure 4). A buffer of 41 m was maintained between SSCDS and airblast sprayer treatment plots. The SSCDS and airblast sprayers were configured to spray at an application rate of 935 L ha^−1^. The spray trials for the airblast sprayer and SSCDS treatments were conducted on two separate days (6 and 9 August 2021). Details of the insecticide application are summarized in Table 2. The choice of pesticides was governed by the standard management practice of the collaborating grower. Twenty‐four hours after spraying, leaves were collected for a leaf bioassay evaluation on OBLR and CM larvae and fruits were collected for a fruit bioassay evaluation on CM larvae. For leaf and fruit sample collection, two trees were randomly selected from each plot in the test treatments. The selected trees were divided vertically into three sampling zones (Figure 5): bottom (0.46–1.53 m a.g.l.), mid (1.53–2.6 m a.g.l.) and top (2.6–3.66 m a.g.l.). Then 192 leaves were collected (two treatments × four plots/treatment × two trees/plot × three zones/tree × four leaves/zone) for the OBLR and CM leaf bioassays, respectively. For the untreated control, 96 leaves were collected from an orchard that had received no pesticide applications for the last 12 months. Some 48 apples (two treatments × four plots/treatment × two trees/plot × three zones/tree × one fruit/zone) were collected from treatment blocks for the fruit CM bioassays. The untreated control used 24 fresh Fuji organic apples that were purchased from a local store. The untreated control apples were further disinfected with ultraviolet light (wavelength, 100–280 nm; power, 5 kJ m^−2^).^46^ The bioassay trials with OBLR were conducted immediately after leaf collection, whereas because of the unavailability of CM larva, leaves and fruits were stored in refrigerator (approximately 4°C) for 14 days before CM larva trials.

**Table 2:** Insecticide mixture applied through both spray systems for biological efficacy evaluation

Chemical	Type	Active ingredient	Application rate (L ha^−1^)	Restricted entry interval (h)
Delegate WG	Insecticide	Spinetoram	0.51	4
Nuprid 4F MAX	Insecticide	Imidacloprid	0.44	12
Cal‐Plex 12	Liquid calcium formulation	Calcium	9.50	0
Phorse	Liquid nutrient mix	Nitrogen/phosphorus pentoxide/potassium oxide	9.50	0

### 2.5 Bioassay preparation

#### 2.5.1 Leaf bioassay

**Figure 6:**
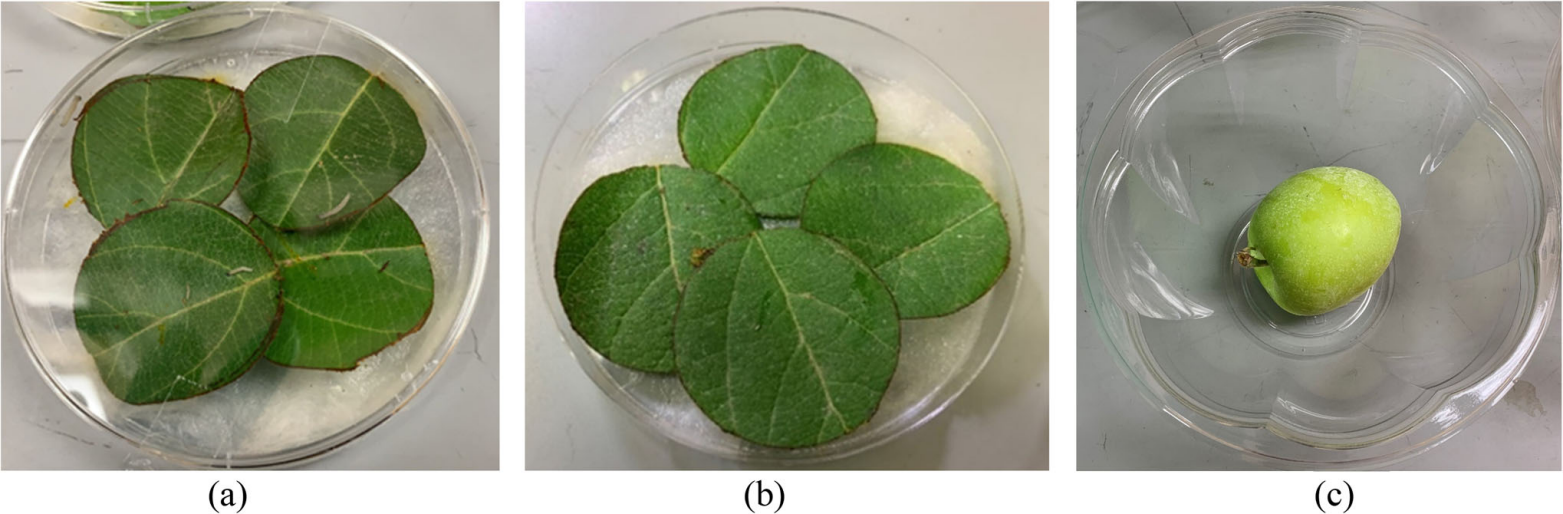
Prepared leaf bioassay arena for (a) obliquebanded leafroller and (b) codling moth as well as (c) fruit bioassay arena for codling moth to investigate the larval mortality of insects

Four leaf disks (4 cm in diameter), one per leaf, were punched and placed on wet cotton with the adaxial surface face up in a 9 cm Petri dish serving as a bioassay arena.^6^, ^36^, ^47^, ^48^ One arena was prepared for each sampling zone, totaling 72 arenas (three treatments [SSCDS, airblast sprayer and untreated control] × four plots/treatment × two trees/plot × three zones/tree × one arena/zone). Five third‐instar OBLR were selected randomly from the rearing cups described above and placed in the bioassay arenas using a fine‐tipped paint brush (Figure 6a). All the arenas were sealed, labeled and stored at room temperature (23 ± 2°C) and a 16:8 h light/dark photoperiod. Larval mortality (Equation 1) was logged after 24 and 48 h of larvae exposure. Similarly, 72 leaf bioassay arenas were prepared for the CM leaf bioassay. Ten first‐instar CM were selected from the wax paper strips and transferred to the arenas using a fine‐tipped paint brush (Figure 6b). The arenas were sealed, labeled and stored under the conditions described above, and larval mortality (Equation 1) was recorded after 24 h.

#### 2.5.2 Fruit bioassay

Plastic bowls (polyethylene terephthalate; top diameter, 17.5 cm; bottom diameter, 8 cm; height, 10 cm; volume, 0.95 L) containing a single fruit were used for the CM fruit bioassay arenas. The experiment consisted of 72 arenas (three treatments [SSCDS, airblast sprayer and untreated control] × four plots/treatment × two trees/plot × three zones/tree × one arena/zone). Five first‐instar CM selected from the rearing apparatus described above were placed directly on the fruits using a fine‐tipped paint brush (Figure 6c). The arena bowls were sealed, labeled and stored at room temperature (23 ± 2°C) and a 16:8 h light/dark photoperiod; lids were perforated as described above. After 7 days of exposure, fruit was dissected and larval mortality was recorded.

### 2.6 Data analysis

The larval mortality (Equation 1) data obtained from the leaf and fruit bioassays were corrected using Abbott's correction formula (Equation 2).^49^ The distribution of the corrected mortality data was not normal and could not be transformed to meet normality assumptions; therefore, a Wilcoxon rank sum test was used to compare larval mortalities between SSCDS and airblast spray treatments. All statistical analysis was performed in R Studio (2021, version 4.0.5)^50^ and results were inferred at the 5% significance level.

(1) $$\text{Mortality}\;\left(\%\right)=\frac{\text{Number}\;\text{of}\;\text{dead}\;\text{larva}\;\text{in}\;\text{arena}}{\text{Total larva in arena}}\times 100$$

(2) $$\text{Corrected mortality}\left(\%\right)=\begin{array}{l} \frac{\%\text{observed mortality}-\%\text{control mortality}}{100-\%\text{control mortality}}\\ \times 100 \end{array}$$

where observed mortality is for SSCDS or airblast sprayer treatment, and control mortality is for untreated control.

## 3 RESULTS

### 3.1 Leaf bioassay

**Figure 7:**
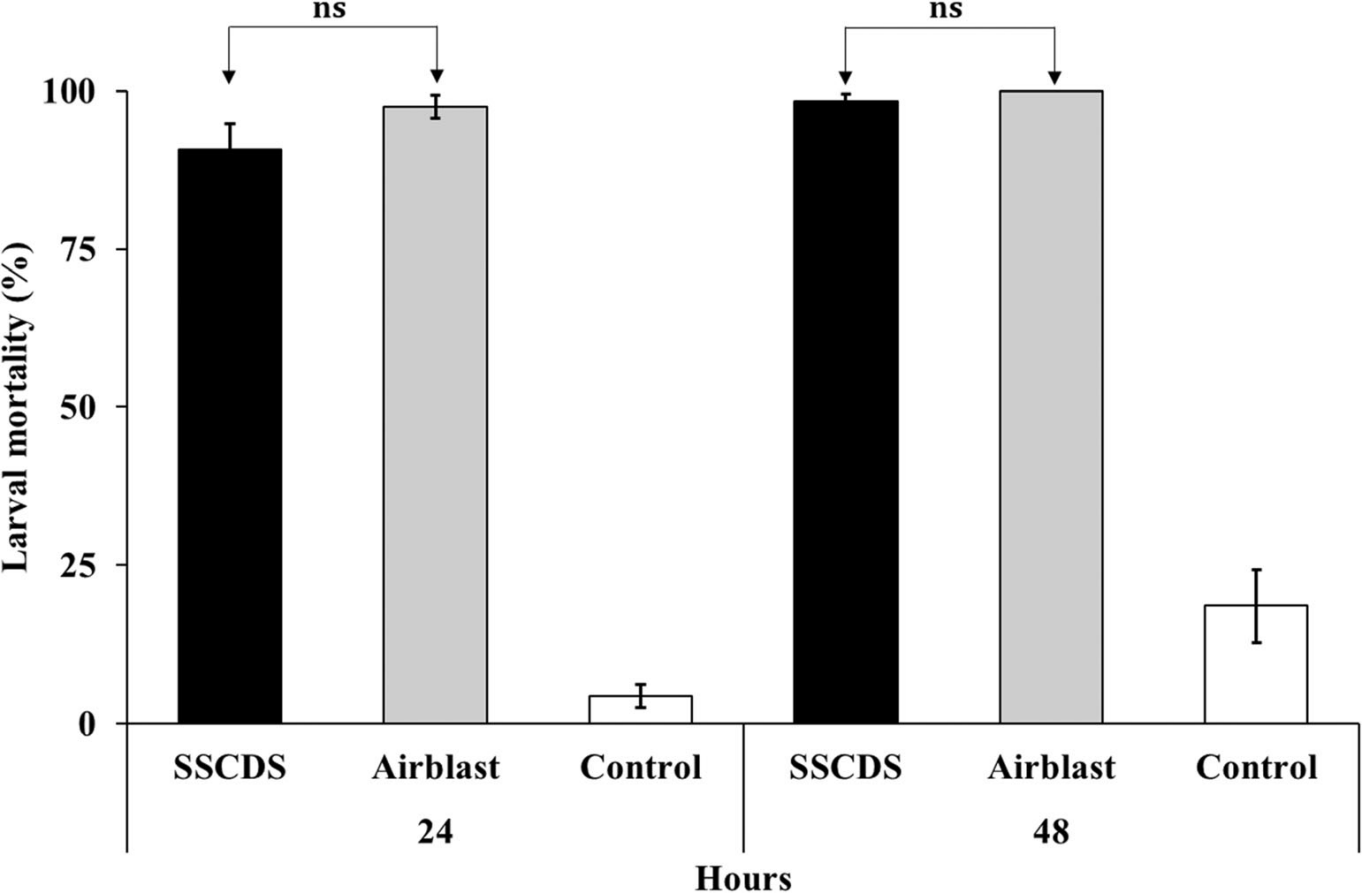
Larval mortality (%) of obliquebanded leafroller on leaves in tested treatments at 24 and 48 h of exposure. Error bars indicate SE; ns, non‐significant difference between treatments based on corrected larval mortality

At 24 h after exposure, the OBLR larval mortalities for SSCDS, airblast sprayer and untreated control treatments were 91%, 98% and 4%, respectively (Figure 7), increasing to 98%, 100% and 19% at 48 h. Post correction, mortality for the SSCDS and airblast sprayer treatments at 24 h after exposure was 90% and 97%, respectively, increasing to 98% and 100% after 48 h. Corrected mortality was not significantly different between SSCDS and the airblast treatment at 24 h (*W* = 336.5, *P* = 0.127) or 48 h (*W* = 312, *P* = 0.162) after exposure.

**Figure 8:**
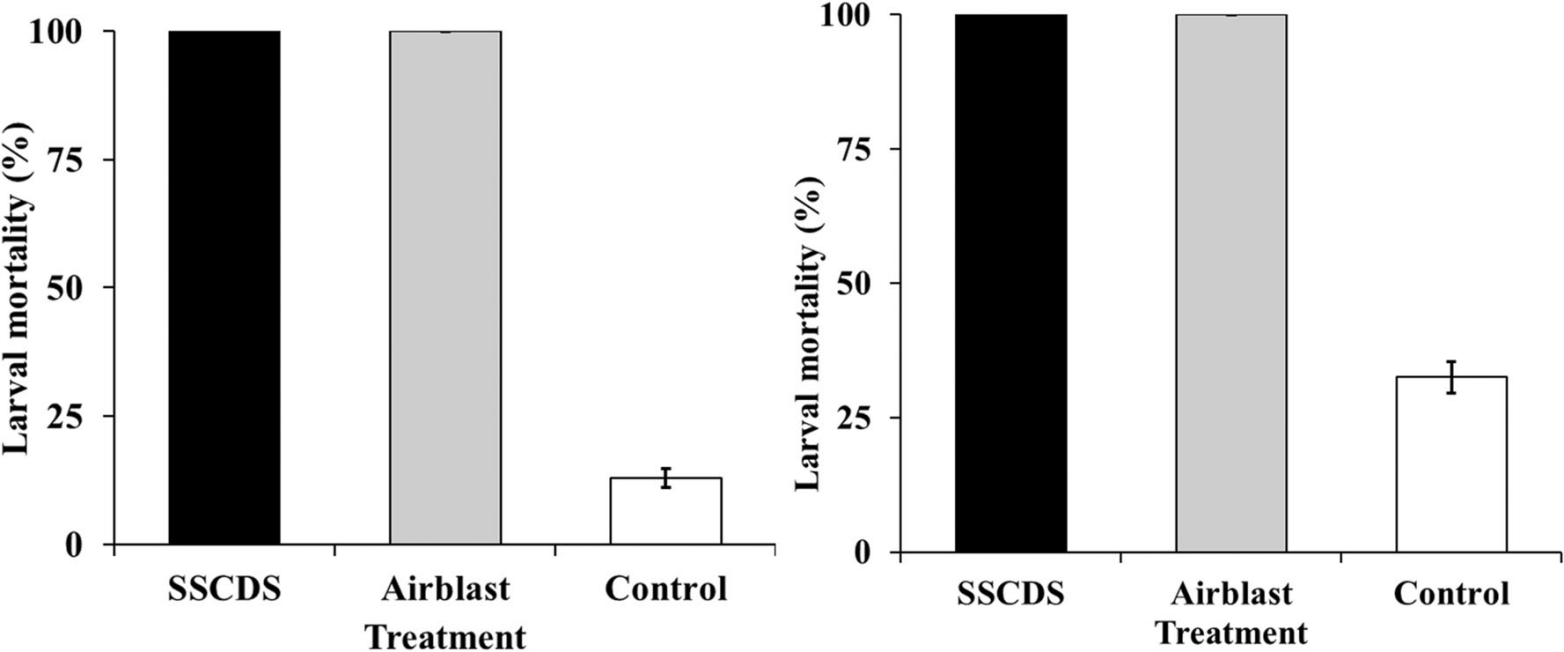
Larval mortality (%) of codling moth on (a) leaves at 24 h exposure and (b) fruits at 7 days exposure for tested treatments. Error bars indicate SE

CM larval mortalities on leaves in the SSCDS and airblast sprayer treatments were 100%, with a control mortality of 13% after 24 h of exposure (Figure 8a). The corrected larval mortality was also 100% for both the spray application treatments.

### 3.2 Fruit bioassay

Like the leaf bioassay, the CM larval mortalities on the fruits for SSCDS and airblast sprayer treatment were 100% after 7 days of exposure (Figure 8b). The respective mortality for untreated control treatment was 33%.

## 4 DISCUSSION

Leaf and fruit bioassay results indicated that SSCDS and airblast sprayer treatments provided comparable larval control for both OBLR and CM. A study from Sahni *et al*. demonstrated that PSD‐based SSCDS provides comparable spray deposition (ng cm^−2^) but significantly lower spray coverage (%) compared with an airblast sprayer. Despite the differences in coverage,^44^ this study confirms that SSCDS has the potential to achieve similar OBLR and CM control as an axial‐fan airblast sprayer. Two bioassay studies conducted in Michigan comparing an SSCDS based on hydraulic spray delivery also demonstrated lower coverage yet near identical disease and pest management compared with an axial‐fan airblast sprayer.^35^, ^36^ In addition, Panneton *et al*. and Verpont *et al*. observed equivalent apple scab control in field studies with severe pest infection testing fixed spray systems despite significantly lower spray coverage compared with an airblast sprayer.^29^, ^30^ These results highlight that achieving pest control is dependent on not only coverage, but a complex of factors including spray coverage, sprayer, pest biology and pesticide active ingredient.^51^ Overall, insect pest and disease management are the ultimate goal of any spray application system. This study indicates that SSCDS can be potentially adopted as an alternative to an airblast sprayer in a high‐value crop with a complex canopy structure such as apple. Future studies will compare season‐long pest management efficacy of PSD‐based SSCDS with airblast sprayer in modern apple orchard and vineyard.

## 5 CONCLUSIONS

This study was conducted to compare the potential pest control efficacy of a PSD‐based SSCDS to that of an axial‐fan airblast sprayer using OBLR and CM as model species. Overall, PSD‐based SSCDS demonstrated similar pest control compared with that of an airblast sprayer treatments in a modern apple orchard trained in tall spindle architecture.

## CONFLICTS OF INTEREST

The authors declare no conflict of interest.

## Data Availability

The data that support the findings of this study are available from the corresponding author upon reasonable request.
